# Diffuse cryptococcosis simulating lung and skin neoplasms

**DOI:** 10.1590/0037-8682-0177-2023

**Published:** 2023-07-24

**Authors:** Raphael de Oliveira e Silva, Paola Isabela Jacobowski

**Affiliations:** 1 Hospital Santa Casa de Misericórdia de Campo Mourão, Campo Mourão, PR, Brasil. Hospital Santa Casa de Misericórdia de Campo Mourão Campo Mourão PR Brasil; 2 Centro Universitário Integrado, Campo Mourão, PR, Brasil. Centro Universitário Integrado Campo Mourão PR Brasil

A 50-year-old man with a history of gastric mucosa-associated lymphoid tissue lymphoma, remained on oncological follow-up when a chest computed tomography (CT) scan (Figure A) revealed an irregular spiculated nodule in the right lung. The patient developed severe headache, vomiting, and pearly skin lesions (Figure B), and thus a recurrent neoplasm with metastases was suspected. Therefore, an incisional biopsy of the skin lesions was performed, along with a cranial CT scan, cerebrospinal fluid (CSF) collection, and routine laboratory tests. The biopsy revealed that the dermis was occupied by round fungal structures surrounded by mucus with sparse lymphocytes and histiocytes. In addition, the CSF collection was positive for Cryptococcus, which was negative on bacterioscopy.

**FIGURE A:**
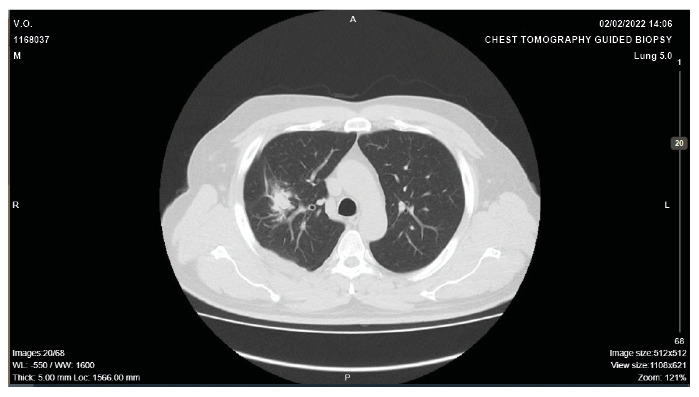
Chest computed tomography with a mediastinal window showing a lesion in the right hemithorax.

**FIGURE B:**
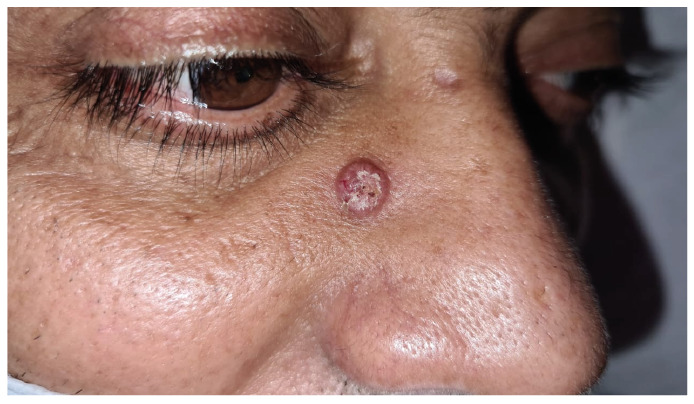
Oval-shaped, scaly, and pearly skin lesion.

Pulmonary cryptococcosis is usually asymptomatic, with the central nervous system and skin being the second and third most common sites of disease dissemination, respectively. The lack of pathognomonic signs and symptoms of the disease, such as cough, fever, hemoptysis, fatigue, and weight loss, along with the absence of diagnostic confirmation via a culture or anatomopathological examination, delay the administration of the adequate treatment. The nodules of pulmonary cryptococcosis may show various tomographic signs, leading to a misdiagnosis of lung cancer, followed by intrapulmonary metastasis or tuberculosis^1^.

Thus, the importance of imaging tests combined with laboratory and histopathological examinations of lesion samples is emphasized, consolidating the correct diagnosis of fungal infection, reinforcing the need for a correct and early diagnosis to prevents the infection’s dissemination and leads to starting treatment as soon as possible^2^^,^^3^.
